# Comparing Random Survival Forests and Cox Regression for Nonresponders to Neoadjuvant Chemotherapy Among Patients With Breast Cancer: Multicenter Retrospective Cohort Study

**DOI:** 10.2196/69864

**Published:** 2025-04-08

**Authors:** Yudi Jin, Min Zhao, Tong Su, Yanjia Fan, Zubin Ouyang, Fajin Lv

**Affiliations:** 1 Department of Radiology The First Affiliated Hospital of Chongqing Medical University Chongqing China; 2 Department of Breast and Thyroid Surgery The First Affiliated Hospital of Chongqing Medical University Chongqing China

**Keywords:** breast cancer, neoadjuvant chemotherapy, pathological complete response, survival risk, random survival forest

## Abstract

**Background:**

Breast cancer is one of the most common malignancies among women worldwide. Patients who do not achieve a pathological complete response (pCR) or a clinical complete response (cCR) post–neoadjuvant chemotherapy (NAC) typically have a worse prognosis compared to those who do achieve these responses.

**Objective:**

This study aimed to develop and validate a random survival forest (RSF) model to predict survival risk in patients with breast cancer who do not achieve a pCR or cCR post-NAC.

**Methods:**

We analyzed patients with no pCR/cCR post-NAC treated at the First Affiliated Hospital of Chongqing Medical University from January 2019 to 2023, with external validation in Duke University and Surveillance, Epidemiology, and End Results (SEER) cohorts. RSF and Cox regression models were compared using the time-dependent area under the curve (AUC), the concordance index (C-index), and risk stratification.

**Results:**

The study cohort included 306 patients with breast cancer, with most aged 40-60 years (204/306, 66.7%). The majority had invasive ductal carcinoma (290/306, 94.8%), with estrogen receptor (ER)+ (182/306, 59.5%), progesterone receptor (PR)– (179/306, 58.5%), and human epidermal growth factor receptor 2 (HER2)+ (94/306, 30.7%) profiles. Most patients presented with T2 (185/306, 60.5%), N1 (142/306, 46.4%), and M0 (295/306, 96.4%) staging (TNM meaning “tumor, node, metastasis”), with 17.6% (54/306) experiencing disease progression during a median follow-up of 25.9 months (IQR 17.2-36.3). External validation using Duke (N=94) and SEER (N=2760) cohorts confirmed consistent patterns in age (40-60 years: 59/94, 63%, vs 1480/2760, 53.6%), HER2+ rates (26/94, 28%, vs 935/2760, 33.9%), and invasive ductal carcinoma prevalence (89/94, 95%, vs 2506/2760, 90.8%). In the internal cohort, the RSF achieved significantly higher time-dependent AUCs compared to Cox regression at 1-year (0.811 vs 0.763), 3-year (0.834 vs 0.783), and 5-year (0.810 vs 0.771) intervals (overall C-index: 0.803, 95% CI 0.747-0.859, vs 0.736, 95% CI 0.673-0.799). External validation confirmed robust generalizability: the Duke cohort showed 1-, 3-, and 5-year AUCs of 0.912, 0.803, and 0.776, respectively, while the SEER cohort maintained consistent performance with AUCs of 0.771, 0.729, and 0.702, respectively. Risk stratification using the RSF identified 25.8% (79/306) high-risk patients and a significantly reduced survival time (*P*<.001). Notably, the RSF maintained improved net benefits across decision thresholds in decision curve analysis (DCA); similar results were observed in external studies. The RSF model also showed promising performance across different molecular subtypes in all datasets. Based on the RSF predicted scores, patients were stratified into high- and low-risk groups, with notably poorer survival outcomes observed in the high-risk group compared to the low-risk group.

**Conclusions:**

The RSF model, based solely on clinicopathological variables, provides a promising tool for identifying high-risk patients with breast cancer post-NAC. This approach may facilitate personalized treatment strategies and improve patient management in clinical practice.

## Introduction

Breast cancer remains one of the most prevalent malignancies among women worldwide, accounting for a significant proportion of cancer-related morbidity and mortality [1,2]. Despite advancements in treatment modalities, including neoadjuvant chemotherapy (NAC), a substantial number of patients do not achieve a complete response (CR). These patients usually have a worse prognosis compared to those who do achieve CR (Multimedia Appendix 1) [3]. This underscores the necessity of developing effective prognostic tools to identify patients at high risk for adverse outcomes. However, there are limited studies focusing on developing predictive models for patients with breast cancer who do not attain a CR following NAC.

Currently, machine learning has emerged as a powerful tool for survival analysis, providing significant advantages over traditional statistical methods [4-6]. Traditional methods use Cox regression to predict the prognosis of patients with cancer. However, it is important to note that if the proportional hazards assumption is violated, the results of the Cox regression model may be biased. Additionally, Cox regression may struggle to capture complex, nonlinear relationships between independent variables and survival time [7]. Furthermore, previous studies have confirmed that some other models outperform the Cox regression model. Especially, many studies have shown that the random survival forest (RSF) model can manage high-dimensional data, improve the accuracy of survival predictions, and support personalized treatment strategies, which typically yields the best performance [8-13].

This study aimed to develop and validate an RSF model to predict survival risk in patients with breast cancer who fail to achieve a CR after NAC, comparing its performance with traditional Cox regression. We hypothesized that the RSF model would offer a reliable tool for clinicians to stratify patients based on predicted survival risks, ultimately supporting more informed treatment decisions and enhancing patient management outcomes.

## Methods

### Recruitment and Study Design

Patients diagnosed with breast cancer at the First Affiliated Hospital of Chongqing Medical University from January 2019 to 2023 were comprehensively reviewed. We selected patients who underwent NAC for subsequent analysis. After administering 4-8 cycles of NAC, clinicians, radiologists, and pathologists evaluated the treatment response. A clinical complete response (cCR) was defined as the complete disappearance of all tumor lesions, as confirmed by imaging examination, lasting for a minimum of 4 weeks [14]. A pathological complete response (pCR) was defined as the absence of any residual invasive tumor in both the breasts and axillary lymph nodes (ypT0ypN0) [15]. Patients without a cCR or pCR were enrolled in this study. The adjuvant treatment process was determined according to the guidelines outlined by the Chinese Society of Clinical Oncology (CSCO) and the National Comprehensive Cancer Network (NCCN) [16,17]. Pathological assessment was conducted according to the American Society of Clinical Oncology guidelines (Multimedia Appendix 2) [18-20].

Initially, we established an RSF model using the entire patient cohort. Subsequently, we examined the relationship between clinicopathological variables and survival outcomes using univariate and multivariate Cox regression analyses. We then constructed a Cox regression model using the variables selected based on *P*<.05 from the multivariate analysis. Furthermore, the Duke University Breast Cancer dataset [21] and data from the Surveillance, Epidemiology, and End Results (SEER) [22] database were used as validation cohorts.

### Ethical Considerations

This study was conducted in accordance with the principles of the Declaration of Helsinki and was approved by the Ethics Committee of the First Affiliated Hospital of Chongqing Medical University (ID: 2020–59). Compensation and informed consent were waived due to the retrospective nature of the study and the use of deidentified patient data.

### Follow-up

All patients included in this study were interviewed through either outpatient visits or telephone consultations. The follow-up period extended from discharge until April 30, 2024. Disease-free survival (DFS) was selected as the primary metric for assessing the patient survival duration. The DFS was defined as the time from surgery to the occurrence of breast cancer recurrence, the diagnosis of a new primary cancer, or death from any cause, whichever comes first.

### Model Development: RSF and Cox Regression

The RSF model was constructed using the *randomForestSRC* package in R (R Foundation for Statistical Computing). A starting model was trained on the entire cohort. The following hyperparameters were configured: number of trees (ntree), node size, mtry, and independent variables. The model underwent 2 key adjustments: (1) hyperparameter tuning via cross-validation to optimize performance and (2) permutation-based variable importance scoring to identify significant predictors. Following optimization, the final model was built. Additionally, variables selected based on *P*<.05 from the multivariate Cox regression analysis were incorporated into the Cox regression model.

### External Validation

In this study, external validation datasets were obtained from 2 sources: the Duke breast cancer dataset and the SEER dataset. For the Duke dataset, the inclusion criterion was that patients had undergone NAC. The exclusion criteria were (1) any missing or unknown information and (2) patients who achieved a CR to NAC. For the SEER dataset, the inclusion criteria were (1) patients registered in registry 8 and (2) patients diagnosed with a malignant breast tumor. The exclusion criteria were (1) patients with more than 1 primary tumor, (2) any missing information or records marked as “Unknown,” (3) patients who did not undergo NAC, and (4) patients who achieved a CR to NAC. In the Duke dataset, survival time was evaluated as recurrence-free survival (RFS), whereas the SEER dataset used overall survival (OS) as its measure.

The performance of the RSF and the Cox regression model was assessed using the 2 validation cohorts. Key metrics, including the concordance index (C-index), 95% CIs, and the integrated Brier score, were calculated to evaluate the models’ predictive accuracy and calibration. Survival curves were generated using the Kaplan-Meier (K-M) method, stratified by risk group identified using the RSF and the Cox regression model. Additionally, variable importance plots were created to illustrate the contribution of each variable to the models. Finally, the models were validated in the Duke and the SEER dataset.

### Model Performance Validation in Different Molecular Subtypes

We further used the RSF model to assess the performance of various molecular subtypes across different datasets, with the results illustrated through time-dependent receiver operating characteristic (ROC) curves. Patients with different subtypes in these datasets were categorized into high- and low-risk groups based on the model’s predictions. The survival differences between these 2 groups were evaluated using K-M curves.

### Statistical Analysis

We conducted statistical analysis using RStudio (R version 4.4.2). All independent variables were categorized and presented as absolute counts. To compare categorical data, we performed the chi-square test. The survival time was a continuous variable and was presented as the median (IQR). The Cox proportional hazards model was used to identify prognostic factors through both univariate and multivariate analyses. For the development of the RSF model, we used the *randomForestSRC* package, while the *cph* function was used to construct the Cox regression model. The ROC curve and decision curve analysis (DCA) were used to display the performance of the models. Survival analysis was performed using K-M curves, with differences between groups evaluated using the log-rank test. In this study, *P*<.05 was considered indicative of a statistically significant difference.

## Results

### Demographic and Clinical Characteristics

**Figure 1:**
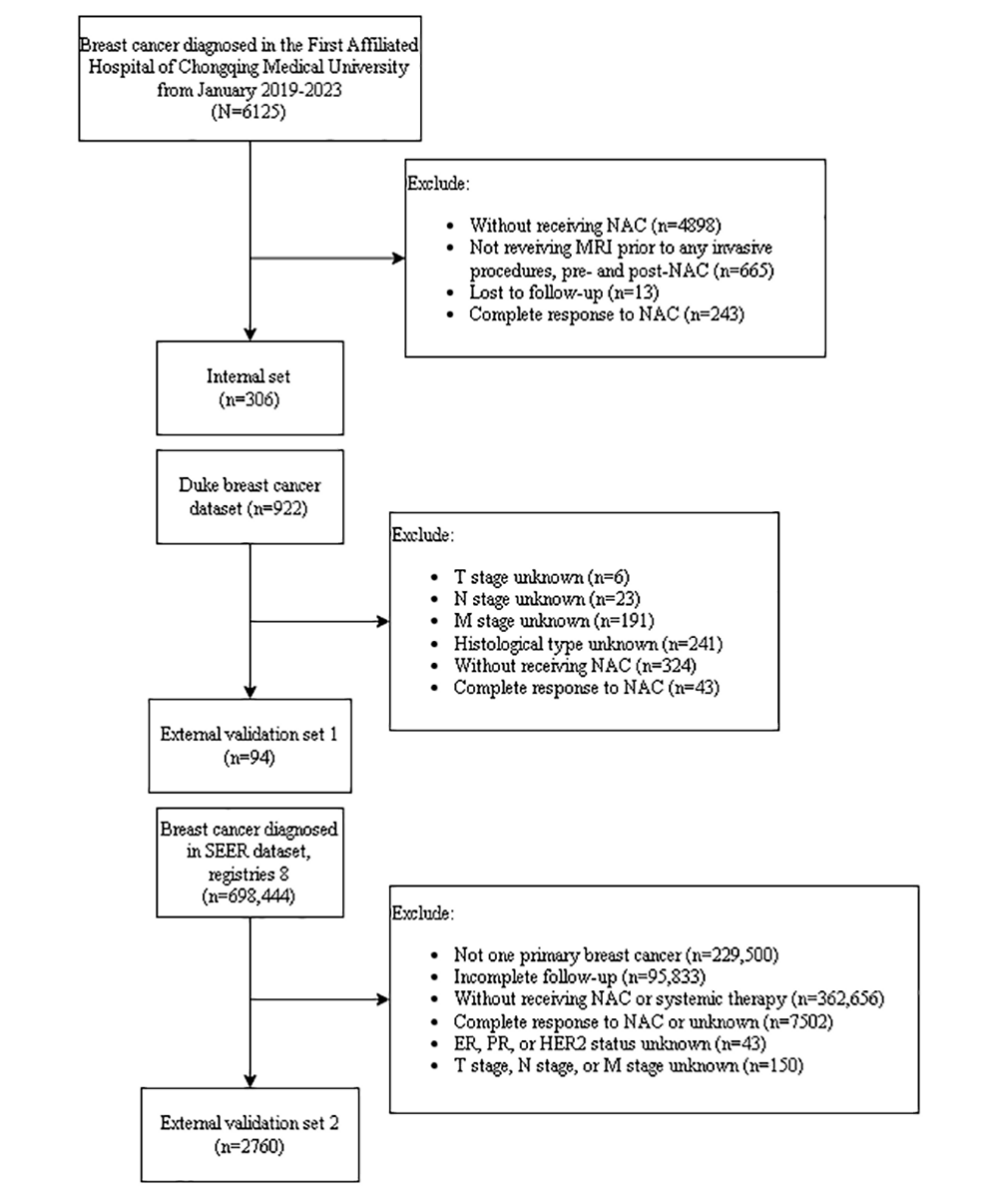
Flowchart of this study. ER: estrogen receptor; HER2: human epidermal growth factor receptor 2; MRI, magnetic resonance imaging; NAC: neoadjuvant chemotherapy; PR: progesterone receptor; TNM: tumor, node, metastasis; SEER: Surveillance, Epidemiology, and End Results.

The study flowchart and patient inclusion process are illustrated in Figure 1. The internal cohort comprised 306 patients with breast cancer with the following characteristics: the majority (204/306, 66.7%) were aged 40-60 years, 94.8% (290/306) had invasive ductal carcinoma, and receptor status analysis revealed 59.5% (182/306) estrogen receptor (ER)+, 58.5% (179/306) progesterone receptor (PR)–, and 30.7% (94/306) human epidermal growth factor receptor 2 (HER2)+ cases. The most prevalent staging was T2 (185/306, 60.5%), N1 (142/306, 46.4%), and M0 (295/306, 96.4%). Over a median follow-up of 25.9 months (IQR 17.2-36.3), 17.6% (54/306) patients experienced disease-related events. External validation cohorts demonstrated comparable patterns, with the Duke dataset (N=94) showing a similar age distribution (59/94, 63%, aged 40-60 years), HER2-positivity rate (26/94, 28%), and hormone receptor (HR) profiles, while the SEER cohort (N=2760) maintained consistent invasive ductal carcinoma predominance (2506/2760, 90.8%) and staging trends. Full demographic details are presented in Table 1.

**Table 1:** Baseline characteristics of patients in the internal, Duke, and SEER^a^ datasets.

Characteristics		Internal dataset (N=306)	Duke dataset (N=94)	SEER dataset (N=2760)
**Age** **(years), n (%)**				
	>60	42 (13.7)	14 (14.9)	732 (26.5)
	≤40	60 (19.6)	21 (22.3)	548 (19.9)
	40-60	204 (66.7)	59 (62.8)	1480 (53.6)
**Histological type, n (%)**				
	Invasive ductal carcinoma	290 (94.8)	89 (94.7)	2506 (90.8)
	Other	16 (5.2)	5 (5.3)	254 (9.2)
**ER^b^, n (%)**				
	Negative	124 (40.5)	40 (42.6)	686 (24.9)
	Positive	182 (59.5)	54 (57.4)	2074 (75.1)
**PR^c^, n (%)**				
	Negative	179 (58.5)	52 (55.3)	1093 (39.6)
	Positive	127 (41.5)	42 (44.7)	1667 (60.4)
**HER2^d^, n (%)**				
	Negative	212 (69.3)	68 (72.3)	1825 (66.1)
	Positive	94 (30.7)	26 (27.7)	935 (33.9)
**Molecular subtype, n (%)**				
	HR^e^–/HER2–	73 (23.9)	29 (30.9)	466 (16.9)
	HR–/HER2+	50 (16.3)	11 (11.7)	182 (6.6)
	HR+/HER2–	139 (45.4)	39 (41.5)	1359 (49.2)
	HR+/HER2+	44 (14.4)	15 (16.0)	753 (27.3)
**T^f^, n (%)**				
	1	36 (11.8)	16 (17.0)	449 (16.3)
	2	185 (60.5)	58 (61.7)	1458 (52.8)
	3	51 (16.7)	17 (18.1)	578 (20.9)
	4	34 (11.1)	3 (3.2)	275 (10.0)
**N^f^, n (%)**				
	0	43 (14.1)	41 (43.6)	929 (33.7)
	1	142 (46.4)	40 (42.6)	1232 (44.6)
	2	61 (19.9)	7 (7.4)	343 (12.4)
	3	60 (19.6)	6 (6.4)	256 (9.3)
**M^f^, n (%)**				
	0	295 (96.4)	93 (98.9)	2677 (97.0)
	1	11 (3.6)	1 (1.1)	83 (3.0)
**S** **tatus** **, n (%)**				
	0	252 (82.4)	83 (88.3)	2364 (85.7)
	1	54 (17.6)	11 (11.7)	396 (14.3)
Survival time, median (IQR)		25.9 (17.2-36.3)	50.2 (29.3-66.2)	35.0 (15.5-64.0)

^a^SEER: Surveillance, Epidemiology, and End Results.

^b^ER: estrogen receptor.

^c^PR: progesterone receptor.

^d^HER2: human epidermal growth factor receptor 2.

^e^HR: hormone receptor.

^f^TNM: tumor, node, metastasis.

### Construction of the RSF Model

**Figure 2:**
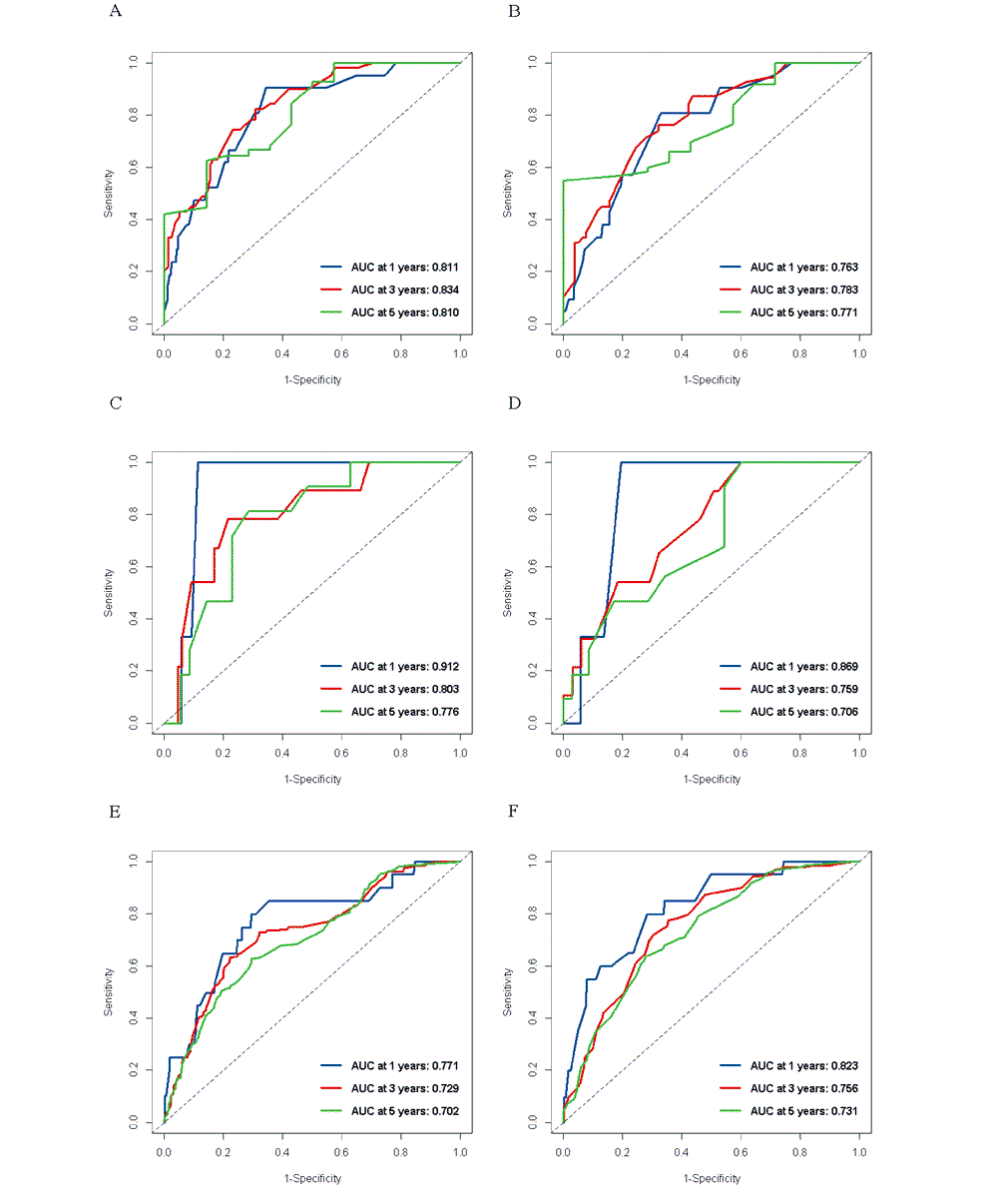
ROC curves of the RSF model in the internal (A), Duke (C), and SEER (E) datasets. ROC curves of the Cox regression model in the internal (B), Duke (D), and SEER (F) datasets. AUC: area under the curve; ROC: receiver operating characteristic; SEER: Surveillance, Epidemiology, and End Results.

Initially, we integrated all independent variables into the RSF model, setting the number of trees (ntree) to 2000. Our findings indicated that the model stabilized when ntree reached 1000. Additionally, variable importance analysis revealed that the variable T stage had significant negative effects on the model’s performance (Multimedia Appendix 3). Consequently, we adjusted ntree to 1000 and included age, histological type, ER, PR, HER2, N stage, and M stage as independent variables to reconstruct the RSF model. Through hyperparameter tuning, we determined that optimal model performance and generalization ability were achieved with a node size of 10 and an mtry of 2 (Multimedia Appendix 4). The ROC curves showed that in the training set, the AUC of the model at 1, 3, and 5 years was 0.811, 0.834, and 0.810, respectively (Figure 2A). The C-index was 0.803 (95% CI 0.747-0.859). The Brier score is shown in Multimedia Appendix 5.

### Construction of the Cox Regression Model

Results of the multivariate Cox regression analysis indicated that age, PR, HER2, N stage, and M stage were significantly associated with survival risks (Table 2). These variables were incorporated into the Cox regression model, which yielded an AUC of 0.763, 0.783, and 0.771 at the 1-, 3-, and 5-year marks, respectively (Figure 2B). The C-index was calculated to be 0.736 (95% CI 0.673-0.799). The Brier score, which was relatively lower than that of the RSF model at each time point, is presented in Multimedia Appendix 5.

**Table 2:** Cox regression of clinicopathological variables.

Variables		Patients (N=306), n (%)	Univariable hazard ratio (95% CI); *P* value	Multivariable hazard ratio (95% CI); *P* value
**Age (years)**				
	>60	42 (13.7)		
	≤40	60 (19.6)	0.36 (0.15-0.87); *P*=.02	0.45 (0.18-1.12); *P*=.09
	40-60	204 (66.7)	0.40 (0.20-0.77); *P*=.01	0.48 (0.24-0.97); *P*=.04
**Histology**				
	Invasive ductal carcinoma	290 (94.8)		
	Other	16 (5.2)	0.31 (0.04-2.22); *P*=.24	0.30 (0.04-2.25); *P*=.24
**ER^a^**				
	Negative	124 (40.5)		
	Positive	182 (59.5)	0.74 (0.44-1.27); *P*=.28	1.05 (0.53-2.09); *P*=.88
**PR^b^**				
	Negative	179 (58.5)		
	Positive	127 (41.5)	0.54 (0.30-0.98); *P*=.04	0.45 (0.21-0.96); *P*=.04
**HER2^c^**				
	Negative	212 (69.3)		
	Positive	94 (30.7)	0.54 (0.29-1.02); *P*=.06	0.45 (0.23-0.87); *P*=.02
**T** ^d^				
	1	36 (11.8)		
	2	185 (60.5)	1.42 (0.55-3.63); *P*=.47	1.47 (0.57-3.82); *P*=.42
	3	51 (16.7)	1.35 (0.44-4.14); *P*=.60	1.26 (0.40-3.93); *P*=.69
	4	34 (11.1)	1.73 (0.56-5.29); *P*=.34	1.58 (0.49-5.08); *P*=.45
**N^d^**				
	0	43 (14.1)		
	1	142 (46.4)	1.84 (0.63-5.38); *P*=.26	1.65 (0.55-4.93); *P*=.37
	2	61 (19.9)	2.75 (0.89-8.55); *P*=.08	2.06 (0.65-6.58); *P*=.22
	3	60 (19.6)	4.34 (1.45-12.96); *P*=.01	3.36 (1.10-10.24); *P*=.03
**M^d^**				
	0	295 (96.4)		
	1	11 (3.6)	3.30 (1.31-8.31); *P*=.01	2.74 (1.03-7.25); *P*=.04

^a^ER: estrogen receptor.

^b^PR: progesterone receptor.

^c^HER2: human epidermal growth factor receptor 2.

^d^TNM: tumor, node, metastasis.

### Model Validation

A total of 94 patients from the Duke dataset and 2760 patients from the SEER dataset were included in the analysis; the selection process is displayed in Figure 1. For the RSF model, the AUC for the Duke dataset was 0.912 at 1 year, 0.803 at 3 years, and 0.776 at 5 years, as illustrated in Figure 2C, while the AUC values for the SEER dataset were 0.771 at 1 year, 0.729 at 3 years, and 0.701 at 5 years, as shown in Figure 2E. For the Cox regression model, the AUC for the Duke dataset was 0.869 at 1 year, 0.759 at 3 years, and 0.706 at 5 years, with the corresponding ROC curves presented in Figure 2D. For the SEER dataset, the AUC values were 0.823 at 1 year, 0.756 at 3 years, and 0.731 at 5 years, as depicted in Figure 2F.

### Comparison of the RSF and Cox Regression Model

Multimedia Appendix 6 presents DCA curves for the 2 models at 1-, 3-, and 5-year intervals across all datasets. In both the training set and the Duke dataset, patients derived greater benefits from the RSF model compared to the Cox regression model. Conversely, in the SEER dataset, the benefits for patients using both models were comparable. Furthermore, in the training set, the RSF model identified a predictive cut-off value of 8.70 to categorize patients into high- and low-risk groups (Multimedia Appendix 7). Survival analysis demonstrated that the prognosis for the high-risk group was significantly worse than that for the low-risk group (Multimedia Appendix 8). This cut-off value was also applied to classify patients from both the Duke and SEER datasets into high- and low-risk groups. Survival analysis indicated that patients in the high-risk group had poorer prognoses compared to those in the low-risk group across both datasets (Multimedia Appendix 8). Similarly, the Cox regression model established a predictive cut-off value of 0.27 in the training set to differentiate between high- and low-risk groups (Multimedia Appendix 7). Survival analysis yielded results consistent with those obtained using the RSF model (Multimedia Appendix 8).

### Performance of the RSF Model Among Different Molecular Subtypes

We conducted a performance evaluation of the RSF model across various molecular subtypes. The ROC curves indicated that in the internal dataset, the RSF model achieved an AUC of 1.000 for both 1- and 3-year survival rates and 0.748 for the 5-year survival rate for the HR+/HER2+ subtype. For the HR+/HER2– subtype, the AUC values were 0.872 for the 1-year, 0.699 for the 3-year, and 0.778 for the 5-year survival rate. For the HR–/HER2+ subtype, the AUC values were 0.639 for the 1-year, 0.845 for the 3-year, and 0.698 for the 5-year survival rate. For the HR–/HER2– subtype, the AUC values were 0.681 for the 1-year and 0.832 for the 3-year survival rate. Consistent trends were observed in the SEER and Duke validation datasets (Multimedia Appendix 9). The K-M curves further demonstrated the RSF model’s ability to stratify patients into distinct high- and low-risk groups across all 4 molecular subtypes in both internal and SEER datasets. In the Duke dataset, although no statistically significant difference was observed in the HR−/HER2− subtype, low-risk patients still exhibited higher RFS compared to high-risk patients (Multimedia Appendix 10).

## Discussion

### Principal Findings

In this study, our key findings demonstrated that the RSF model outperforms Cox regression in predicting survival risk for nonresponders post-NAC, with validated generalizability across external cohorts. The RSF model also demonstrated consistent effectiveness when analyzing various molecular subtypes. The findings highlight the potential of machine learning techniques, particularly the RSF, in enhancing prognostic accuracy and guiding clinical decision-making in oncology.

Previous research has demonstrated that achieving a pCR following NAC is associated with significantly improved event-free survival and OS [3]. Consequently, numerous studies have concentrated on predicting tumor responses to NAC. For instance, Zhao et al [23] constructed machine learning models to predict the pCR to NAC based on clinicopathological variables. Similarly, Zhang and coworkers [24-30] developed machine learning models that incorporated clinicopathological features, radiomic features, and pathomic features to forecast the pCR following NAC. Additionally, Sammut et al [31] and Chen et al [32] created models using multi-omics data. These studies have highlighted the substantial value of machine learning models in predicting patients’ responses to NAC.

However, as previous research has confirmed that patients with no pCR tend to have a worse prognosis and a higher risk for adverse events, it is important to note that few studies have explored risk stratification among those patients. In one of our earlier studies, we tried to develop a random forest model to predict event occurrence among patients with breast cancer with no response to NAC [33]. At the same time, we aimed to create a model that directly predicted patients’ survival risk. The RSF algorithm integrates random forests with survival analysis, enabling the prediction of individual event probabilities and survival time. Compared to the traditional Cox regression, the RSF model offers several advantages: It is not limited by the proportional hazards assumption, it can effectively handle high-dimensional data, and it demonstrates strong generalization capabilities [11]. Liao et al [9] developed a prediction model using 10 machine learning algorithms across 101 combinations to forecast cancer-related mortality in patients with gastric neuroendocrine neoplasms. They finally found that the RSF model obtains the highest AUC value [9]. Similar results have been found in other studies [8,10-13]. In our study, we also found that the RSF model outperforms the Cox regression model, a finding that was further validated using the Duke dataset.

### Strengths

One of the strengths of our study is the robust validation of the RSF model using 2 independent validation cohorts. The C-index demonstrated the model’s predictive accuracy, suggesting that it can reliably stratify patients based on their survival risk. Additionally, the survival time metrics varied across datasets: the training set used DFS, whereas the Duke dataset was based on the RFS, and the SEER dataset used OS. The DFS status encompassed disease recurrence, new primary diseases, and death. Consequently, the model demonstrated strong adaptability across datasets that use these endpoints as status indicators. Therefore, we suggested using DFS to construct time-to-event predictive models in future studies. Another issue pertains to the selection of independent variables. Initially, we tried to develop the RSF model using variables selected through multivariate analysis in the Cox regression; however, the results were not satisfactory. Subsequently, we used the variable importance metrics from the RSF algorithm to identify variables that were ultimately included in the model. Hence, we also suggested using a variable selection method that aligns with the specific model being used.

### Limitations

To the best of our knowledge, our study demonstrated that a more complex algorithm could effectively predict the survival risk of patients with breast cancer without a CR post-NAC, based solely on clinicopathological variables. Nevertheless, there are limitations of our study. First, the sample of the training set was relative small, which might affect variable selection and the parameters set. Second, the follow-up duration in our study was relatively short, with only a limited number of patients followed for more than 5 years. This might have constrained the models’ ability to accurately predict long-term prognosis. Third, the retrospective nature of the data collection might introduce biases, and the findings should be interpreted with caution. Fourth, we included only a limited subset of clinicopathological variables, and further exploration is needed to assess the potential inclusion of additional variables. Additionally, we noted that the performance of the 2 models was comparable in the SEER dataset, with the Cox regression model performing slightly better. This might be attributed to the limited training sample size, which could restrict the performance of the RSF model. Nevertheless, our findings confirm the potential of the RSF model for predicting prognosis using clinicopathological variables in patients. Further prospective study is necessary to confirm its applicability. Moreover, the RSF model is particularly well suited for handling higher-dimensional variables. Thus, future studies should also explore the integration of radiomics, pathomics, and molecular data to enhance the predictive power of the model [34-36].

### Conclusion

Our study highlighted the feasibility and effectiveness of using an RSF model based exclusively on clinicopathological variables to predict survival risk in patients with breast cancer who do not achieve a CR after NAC. This approach could enhance clinical practice by assisting oncologists in identifying high-risk patients who might benefit from more aggressive treatment strategies or closer monitoring. As we continue to refine and validate this model, we anticipate that it would significantly contribute to the advancement of personalized medicine in breast cancer care.

## Appendix

Multimedia Appendix 1 Survival analysis comparing patients who achieved a pCR or a cCR after NAC with those who did not, using the internal dataset across 4 groups: (A) all patients, (B) HR+ patients, (C) HR–/HER2+ patients, and (D) HR–/HER2– patients. cCR: clinical complete response; HER2: human epidermal growth factor receptor 2; HR: hormone receptor; NAC: neoadjuvant chemotherapy; pCR: pathological complete response.

Multimedia Appendix 2 Treatment protocols and pathological assessment of the patients in the internal dataset.

Multimedia Appendix 3 (A) Relationship between the number of trees in the model and the OOB error rate, and (B) relative contribution of each predictor variable to the model’s overall predictive accuracy. OOB: out of bag.

Multimedia Appendix 4 OOB error rate for node size and mtry selection. OOB: out of bag.

Multimedia Appendix 5 Brier scores of the RSF and Cox regression models. RSF: random survival forest.

Multimedia Appendix 6 DCA curves presented for 1-, 3-, and 5-year intervals for the internal set (A-C), the Duke dataset (D-F), and the SEER dataset (G-I). DCA: decision curve analysis; SEER: Surveillance, Epidemiology, and End Results.

Multimedia Appendix 7 Risk group setting using RSF (A) and Cox regression (B) models. RSF: random survival forest.

Multimedia Appendix 8 Stratification of patients into high- and low- groups across the internal (A), Duke (C), and SEER (E) datasets using the RSF model and across the internal (B), Duke (D), and SEER (F) datasets using the Cox regression model. RSF: random survival forest; SEER: Surveillance, Epidemiology, and End Results.

Multimedia Appendix 9 ROC curves for HR+/HER2+ (A), HR+/HER2– (B), HR–/HER2+ (C), and HR–/HER2– (D) subtypes were analyzed at 1-, 3-, and 5-year time points in the internal dataset, with the exception of the HR–/HER2– subtype, which was assessed only at 1- and 3-year time points. In the SEER dataset, ROC curves for HR+/HER2+ (E), HR+/HER2– (F), HR–/HER2+ (G), and HR–/HER2– (H) subtypes were analyzed at 1-, 3-, and 5-year time points, except for the HR–/HER2+ subtype, which was evaluated only at 3- and 5-year time points. The Duke dataset included ROC curves for HR+/HER2– (I) and HR–/HER2– (J) subtypes at all 3 time points (1, 3, and 5 years). (Owing to the limited number of HR+/HER2+ and HR–/HER2+ subtypes in the Duke dataset, ROC curves could not be plotted.）HER2: human epidermal growth factor receptor 2; HR: hormone receptor; ROC: receiver operating characteristic; SEER: Surveillance, Epidemiology, and End Results.

Multimedia Appendix 10 K-M curves of the RSF model stratified patients with HR+/HER2+, HR+/HER2–, HR–/HER2+, and HR–/HER2– subtypes into high- and low-risk groups within the internal (A-D, respectively) and SEER (E-H, respectively) datasets. Similarly, the RSF model divided patients with HR+/HER2– and HR–/HER2– subtypes into high and low-risk groups within the Duke dataset (I-J, respectively). (Owing to the limited number of HR+/HER2+ and HR–/HER2+ subtypes in the Duke dataset, K-M curves could not be plotted.）HER2: human epidermal growth factor receptor 2; HR: hormone receptor; K-M: Kaplan-Meier; RSF: random survival forest; SEER: Surveillance, Epidemiology, and End Results.

## Abbreviations

AUC: area under the curve
cCR: clinical complete response
C-index: concordance index
CR: complete response
DCA: decision curve analysis
DFS: disease-free survival
ER: estrogen receptor
HER2: human epidermal growth factor receptor 2
HR: hormone receptor
K-M: Kaplan-Meier
NAC: neoadjuvant chemotherapy
OS: overall survival
pCR: pathological complete response
PR: progesterone receptor
RFS: recurrence-free survival
ROC: receiver operating characteristic
RSF: random survival forest
SEER: Surveillance, Epidemiology, and End Results
TNM: tumor, node, metastasis

## Data Availability

The data of the First Affiliated Hospital of Chongqing Medical University are available from the corresponding author upon request. The data of the Duke breast cancer dataset can be searched online [21]. The data of the SEER dataset can be retrieved from SEERStat [22].
