# Supplementary figures and images for: Comparing Random Survival Forests and Cox Regression for Nonresponders to Neoadjuvant Chemotherapy Among Patients With Breast Cancer: Multicenter Retrospective Cohort Study
Source: J Med Internet Res. 2025 Apr 8;27:e69864. doi: 10.2196/69864 (PMC12015342; doi:10.2196/69864)

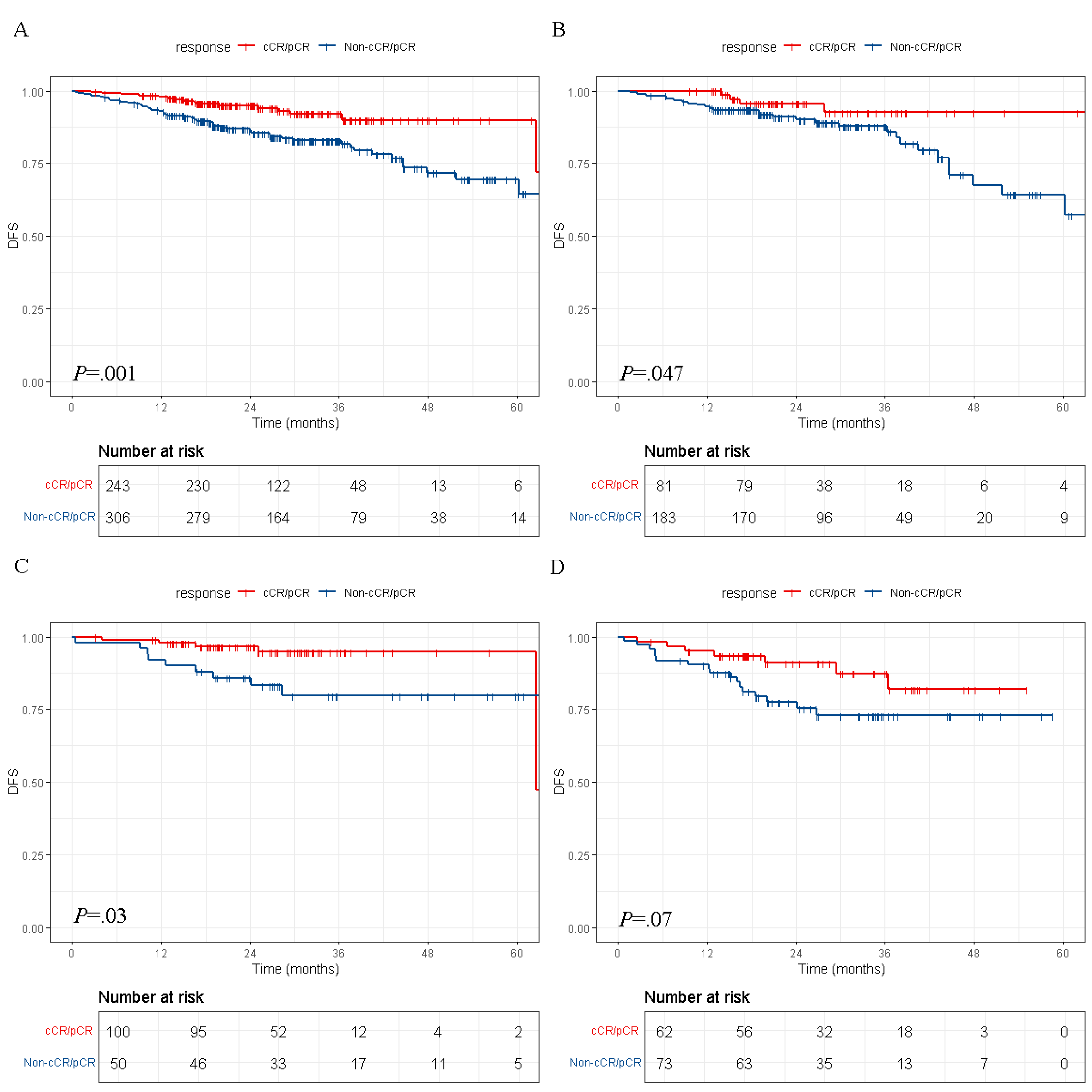

Supplement: Multimedia Appendix 1 [file jmir_v27i1e69864_app1.png]

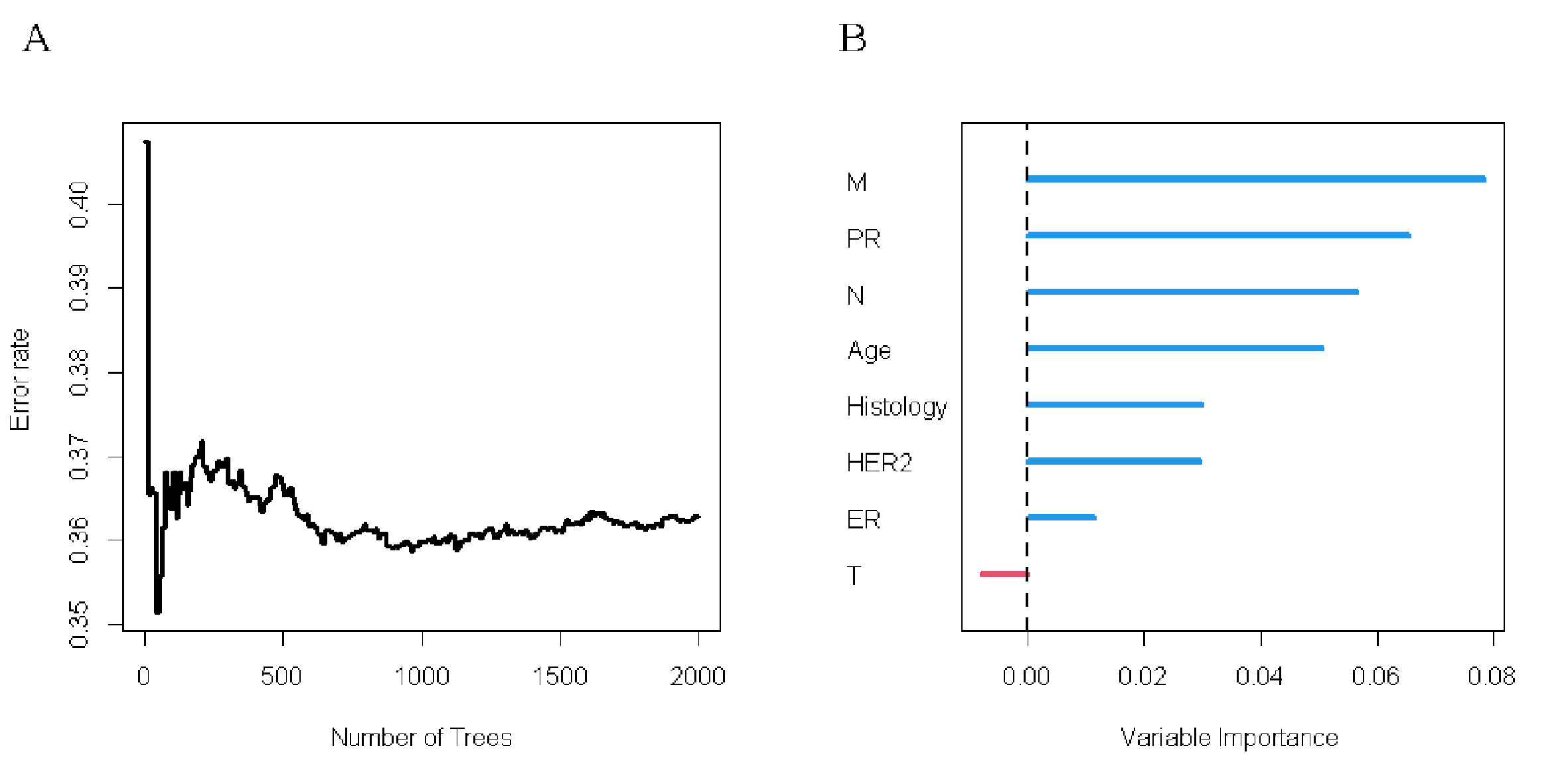

Supplement: Multimedia Appendix 3 [file jmir_v27i1e69864_app3.png]

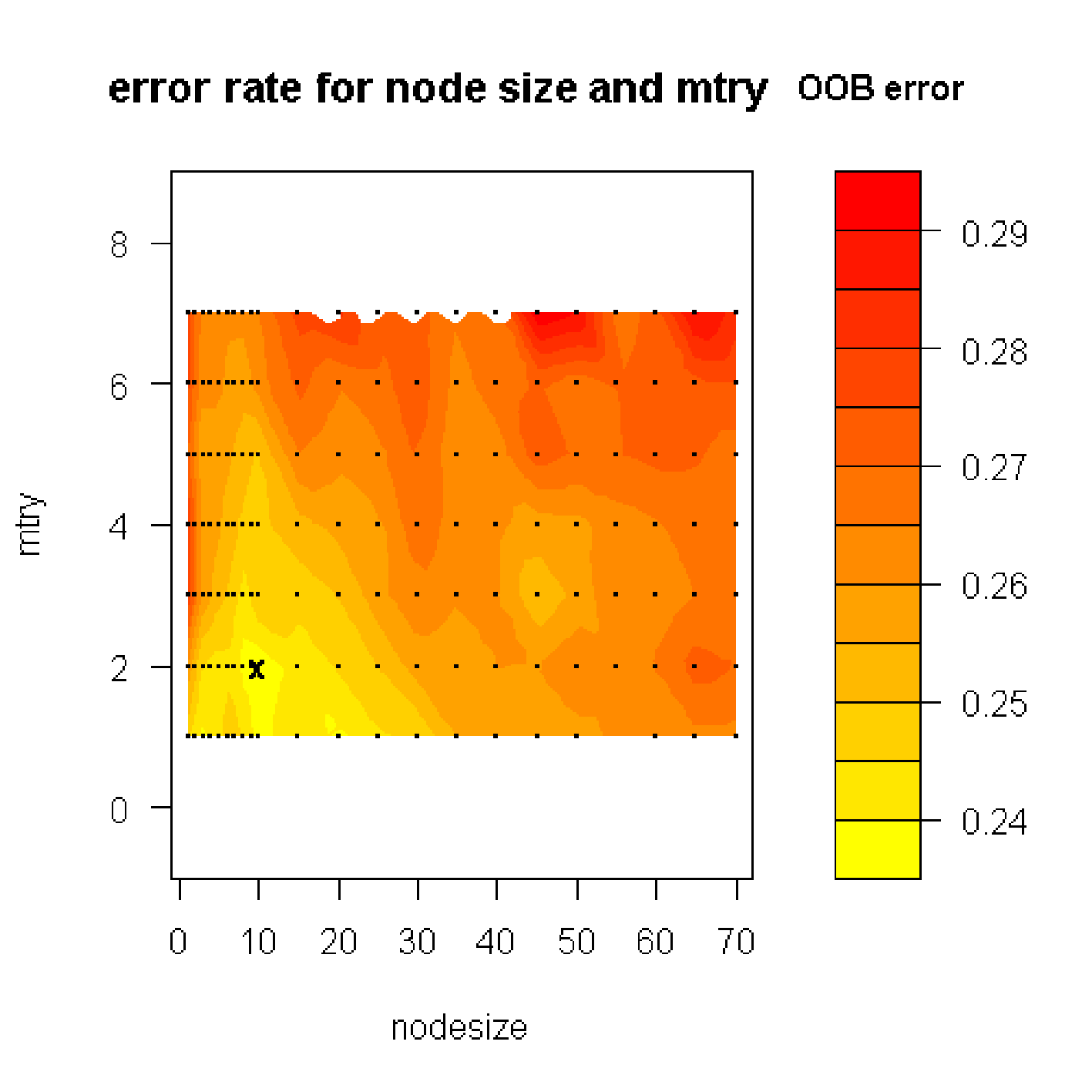

Supplement: Multimedia Appendix 4 [file jmir_v27i1e69864_app4.png]

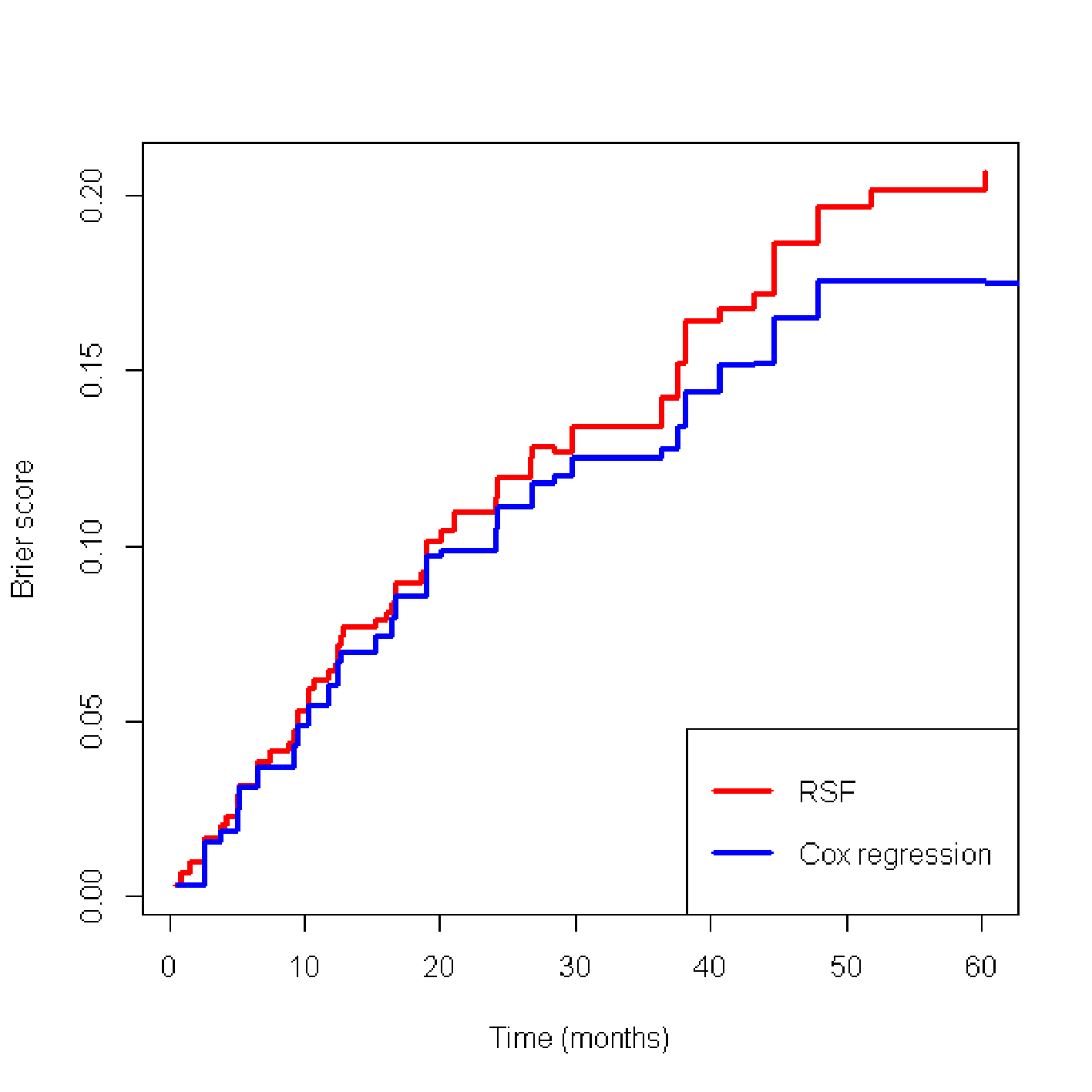

Supplement: Multimedia Appendix 5 [file jmir_v27i1e69864_app5.png]

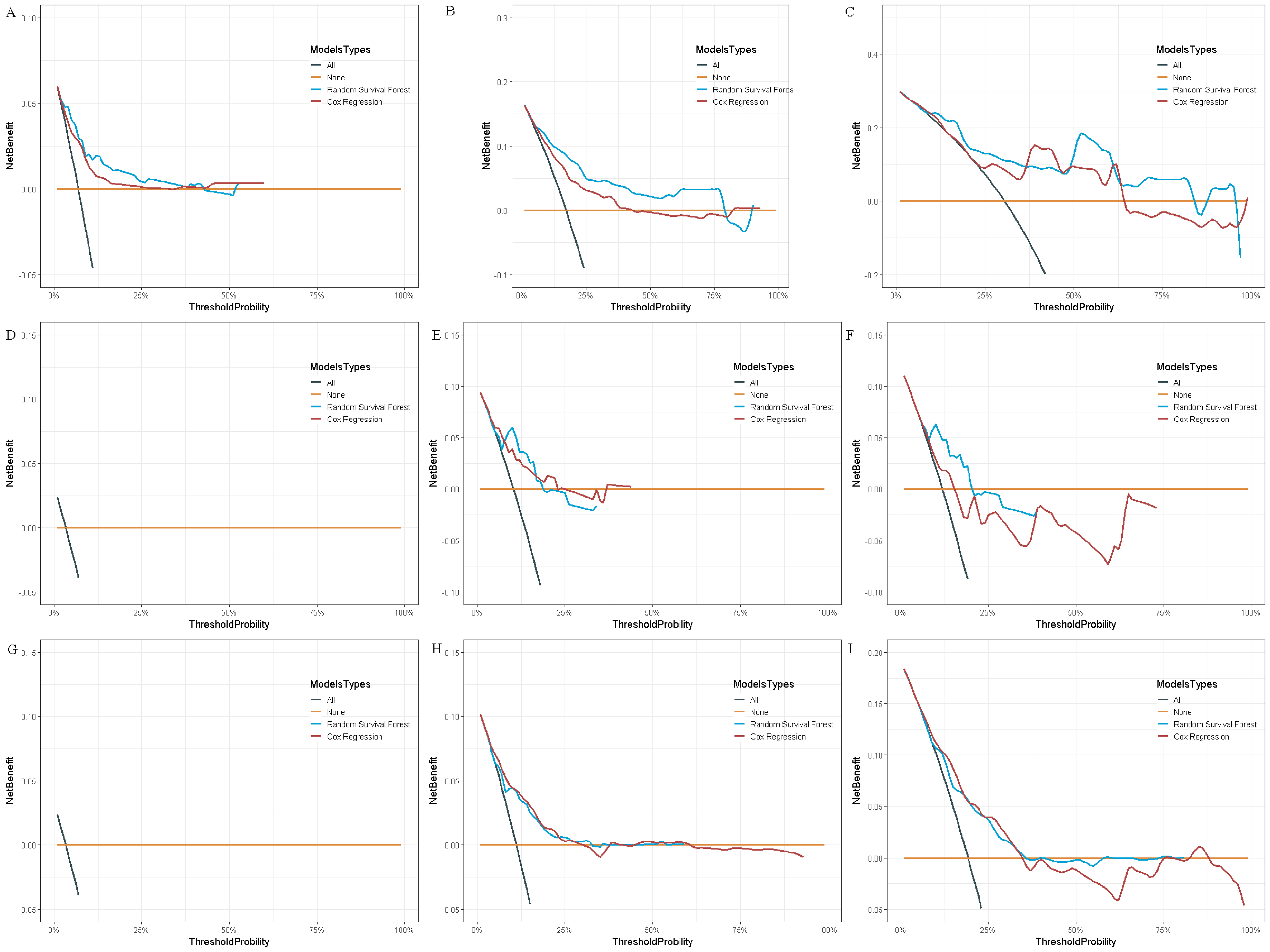

Supplement: Multimedia Appendix 6 [file jmir_v27i1e69864_app6.png]

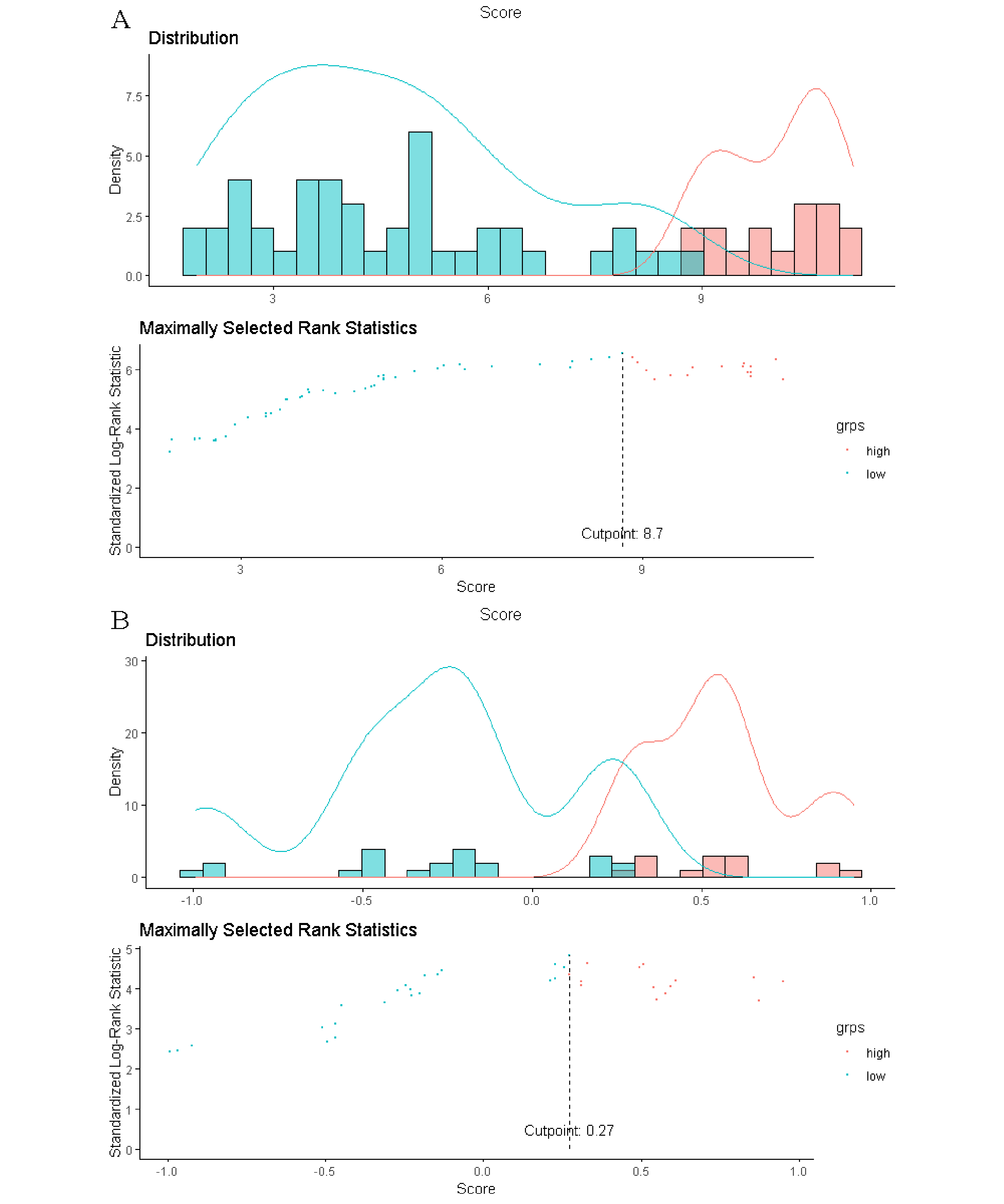

Supplement: Multimedia Appendix 7 [file jmir_v27i1e69864_app7.png]

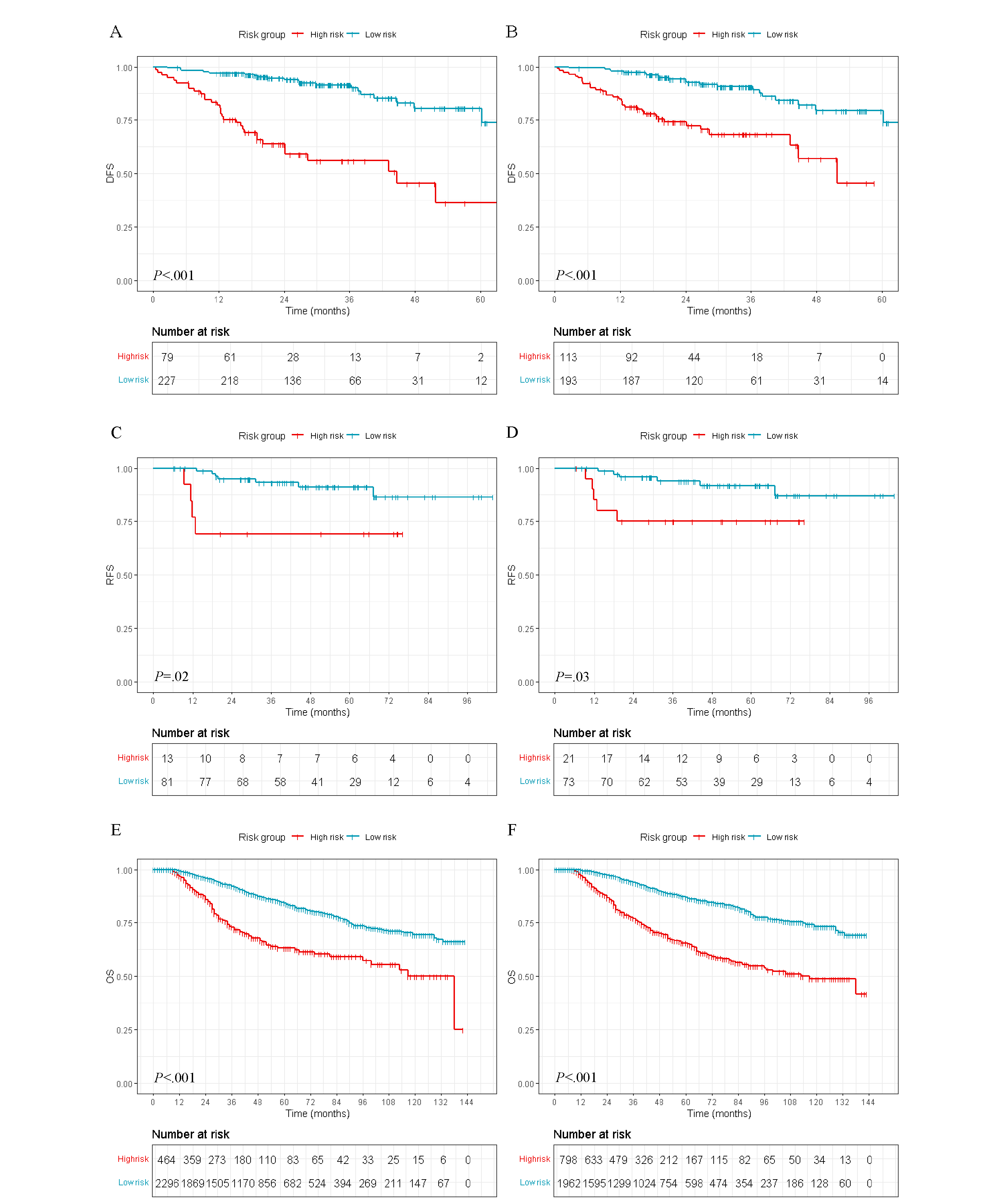

Supplement: Multimedia Appendix 8 [file jmir_v27i1e69864_app8.png]

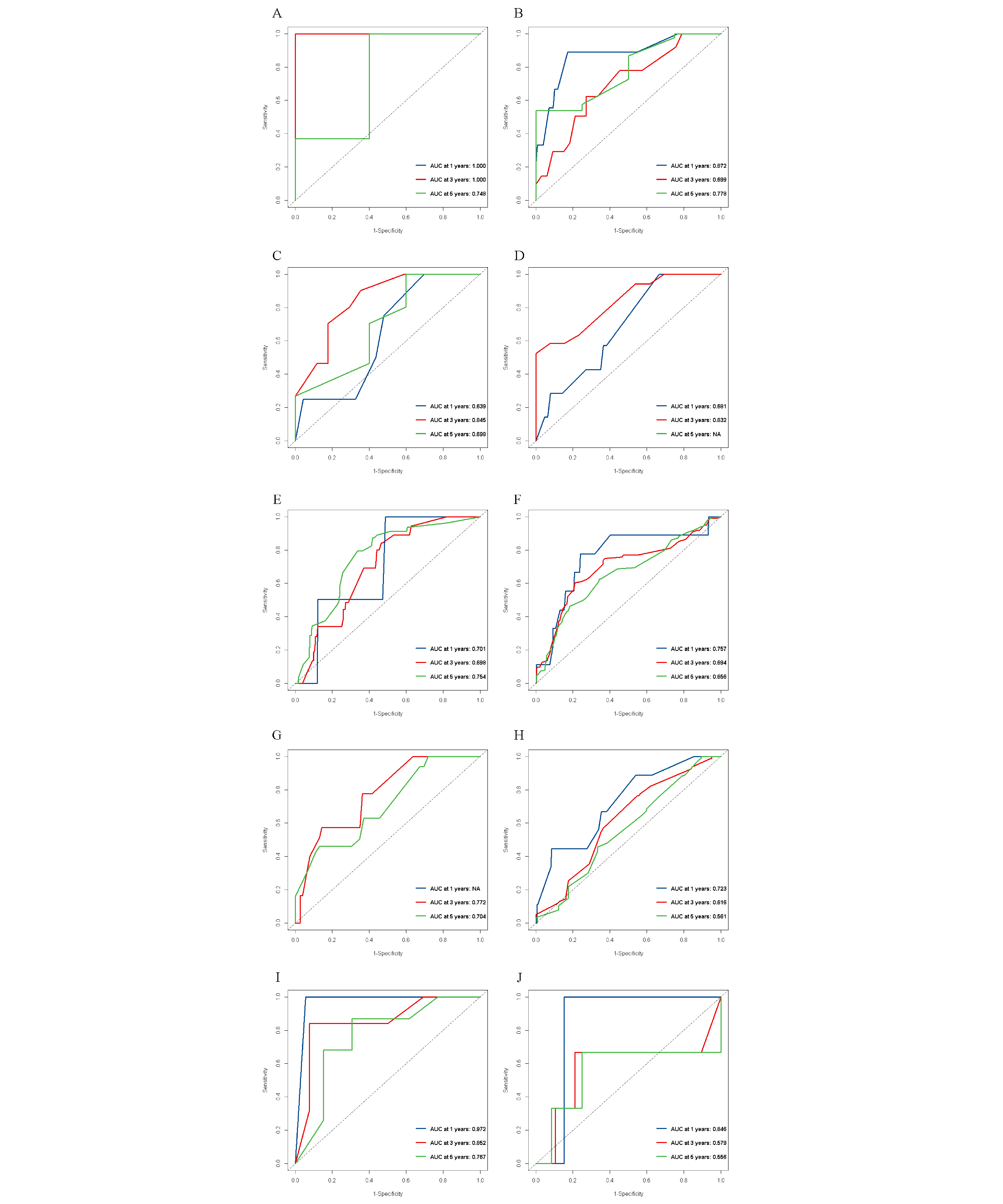

Supplement: Multimedia Appendix 9 [file jmir_v27i1e69864_app9.png]

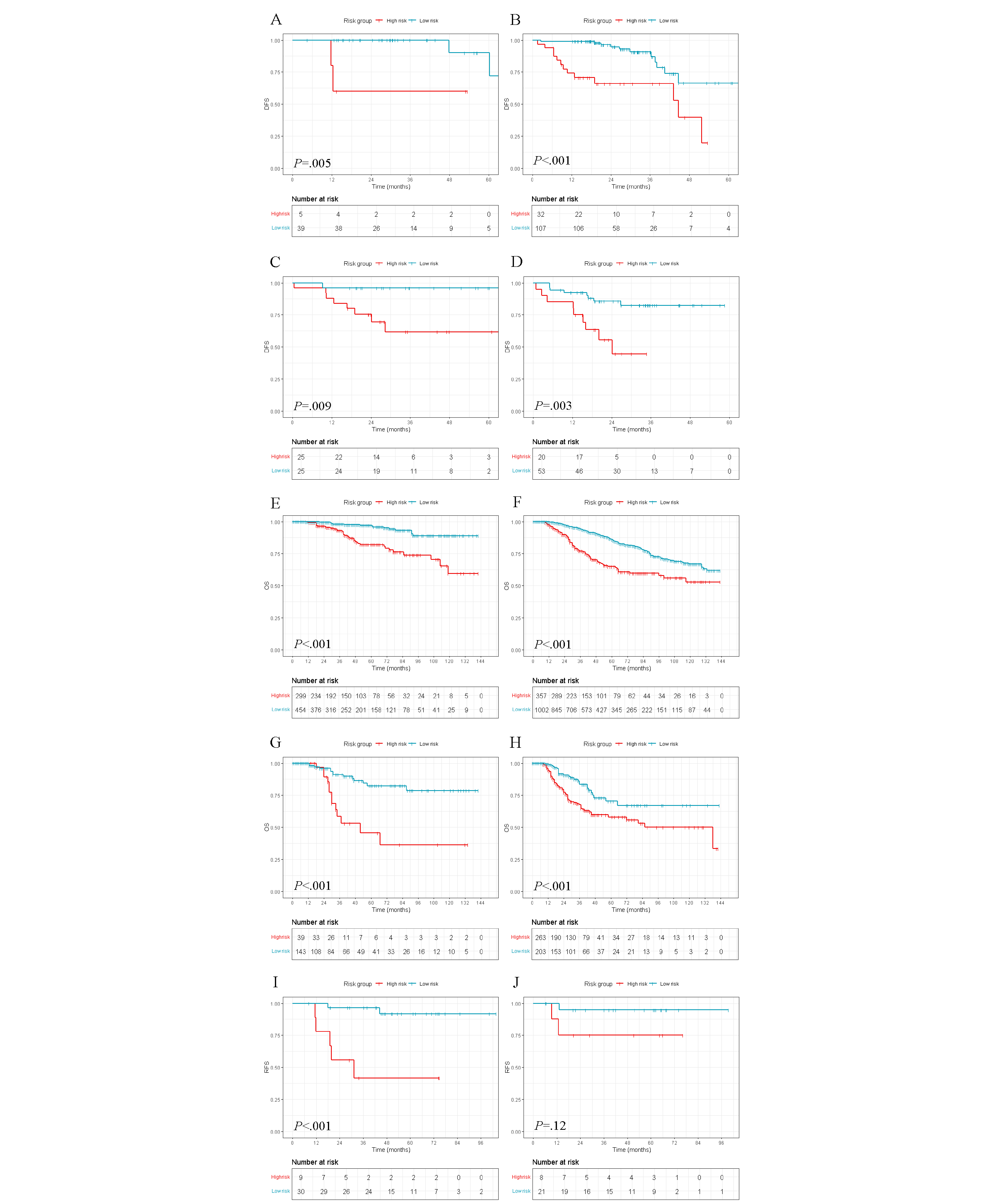

Supplement: Multimedia Appendix 10 [file jmir_v27i1e69864_app10.png]
