# Supplementary material for: Comparing Random Survival Forests and Cox Regression for Nonresponders to Neoadjuvant Chemotherapy Among Patients With Breast Cancer: Multicenter Retrospective Cohort Study
Source: J Med Internet Res. 2025 Apr 8;27:e69864. doi: 10.2196/69864 (PMC12015342; doi:10.2196/69864)
Supplement: Multimedia Appendix 2 [file jmir_v27i1e69864_app2.docx]

### Treatment Protocols

Patients who met the criteria were scheduled to begin NAC within a week. The treatment protocol was mainly TEC (docetaxel 75 mg/m2, epirubicin 75 mg/m^2^, and cyclophosphamide 500 mg/m^2^), TCbHP (docetaxel 75 mg/m^2^; trastuzumab, a loading dose of 8 mg/kg with a maintenance dose of 6 mg/kg; and pertuzumab, a loading dose of 840 mg with a maintenance dose of 420 mg), or THP (docetaxel 75 mg/m^2^; carboplatin, area under curve = 6; trastuzumab, a loading dose of 8 mg/kg with a maintenance dose of 6 mg/kg; and pertuzumab, a loading dose of 840 mg with a maintenance dose of 420 mg). The drugs were administered in 21-day cycles. Following completion of NAC, either mastectomy or breast-conserving surgery along with axillary lymphadenectomy was conducted. Additionally, the systemic therapy procedures were as follows: Patients positive for estrogen receptor (ER) or progesterone receptor (PR) required hormone therapy. Adjuvant Chemotherapy: The indications for postoperative chemotherapy include (1) A relatively larger tumor size; (2) axillary lymph node positivity; (3) Triple-negative breast cancer; (4) HER2-positive status; (5) histological grade III; and (6) elevated Ki-67 proliferation index; (7) Genetic testing indicating a high risk of recurrence. Post-Mastectomy Radiation Therapy (PMRT): For patients with node-positive disease, all individuals will receive PMRT to the chest wall. In contrast, for node-negative patients with tumors measuring 5 cm or less and clear margins (≥ 1 mm), PMRT is generally not required. However, for high-risk patients, including those with: (1) Medially or centrally located tumors. (2) T3-stage tumor. (3) Tumors larger than 2 cm with fewer than 10 axillary nodes removed. (4) At least one of the following characteristics: (a) grade 3, (b) ER-negative, or (c) lymphovascular invasion, PMRT should be considered.

### Pathological Assessment

The immunohistochemistry (IHC) index was evaluated based on core needle biopsy samples of the tumor. Hormone receptor (HR) positivity was defined as the percentage of cells expressing ER or PR being greater than 10% as determined by IHC. HER2 status was classified according to the IHC score and the results of fluorescence in situ hybridization (FISH). A score of 0, 1+, or 2+ without amplification of the ERBB2 gene was considered HER2 negative, while a score of 3+ or 2+ with ERBB2 gene amplification was classified as HER2 positive. These evaluations were conducted independently and blindly by two pathologists.
